# Calcium Induced Regulation of Skeletal Troponin — Computational Insights from Molecular Dynamics Simulations

**DOI:** 10.1371/journal.pone.0058313

**Published:** 2013-03-15

**Authors:** Georgi Z. Genchev, Tomoyoshi Kobayashi, Hui Lu

**Affiliations:** 1 Bioinformatics Program, Department of Bioengineering, University of Illinois at Chicago, Chicago, Illinois, United States of America; 2 Department of Physiology and Biophysics and Center for Cardiovascular Research, University of Illinois at Chicago, Chicago, Illinois, United States of America; 3 Shanghai Institute of Medical Genetics, Children’s Hospital of Shanghai, Shanghai, China; 4 Key Lab of Embryo Molecular Biology, Ministry of Health, Shanghai, China; 5 Shanghai Lab of Embryo and Reproduction Engineering, Shanghai, China; Jr,; Wake Forest University, United States of America

## Abstract

The interaction between calcium and the regulatory site(s) of striated muscle regulatory protein troponin switches on and off muscle contraction. In skeletal troponin binding of calcium to sites I and II of the TnC subunit results in a set of structural changes in the troponin complex, displaces tropomyosin along the actin filament and allows myosin-actin interaction to produce mechanical force. In this study, we used molecular dynamics simulations to characterize the calcium dependent dynamics of the fast skeletal troponin molecule and its TnC subunit in the calcium saturated and depleted states. We focused on the N-lobe and on describing the atomic level events that take place subsequent to removal of the calcium ion from the regulatory sites I and II. A main structural event - a closure of the A/B helix hydrophobic pocket results from the integrated effect of the following conformational changes: the breakage of H-bond interactions between the backbone nitrogen atoms of the residues at positions 2, 9 and sidechain oxygen atoms of the residue at position 12 (N^2^-OE^12^/N^9^-OE^12^) in sites I and II; expansion of sites I and II and increased site II N-terminal end-segment flexibility; strengthening of the β-sheet scaffold; and the subsequent re-packing of the N-lobe hydrophobic residues. Additionally, the calcium release allows the N-lobe to rotate relative to the rest of the Tn molecule. Based on the findings presented herein we propose a novel model of skeletal thin filament regulation.

## Introduction

**Figure 1:**
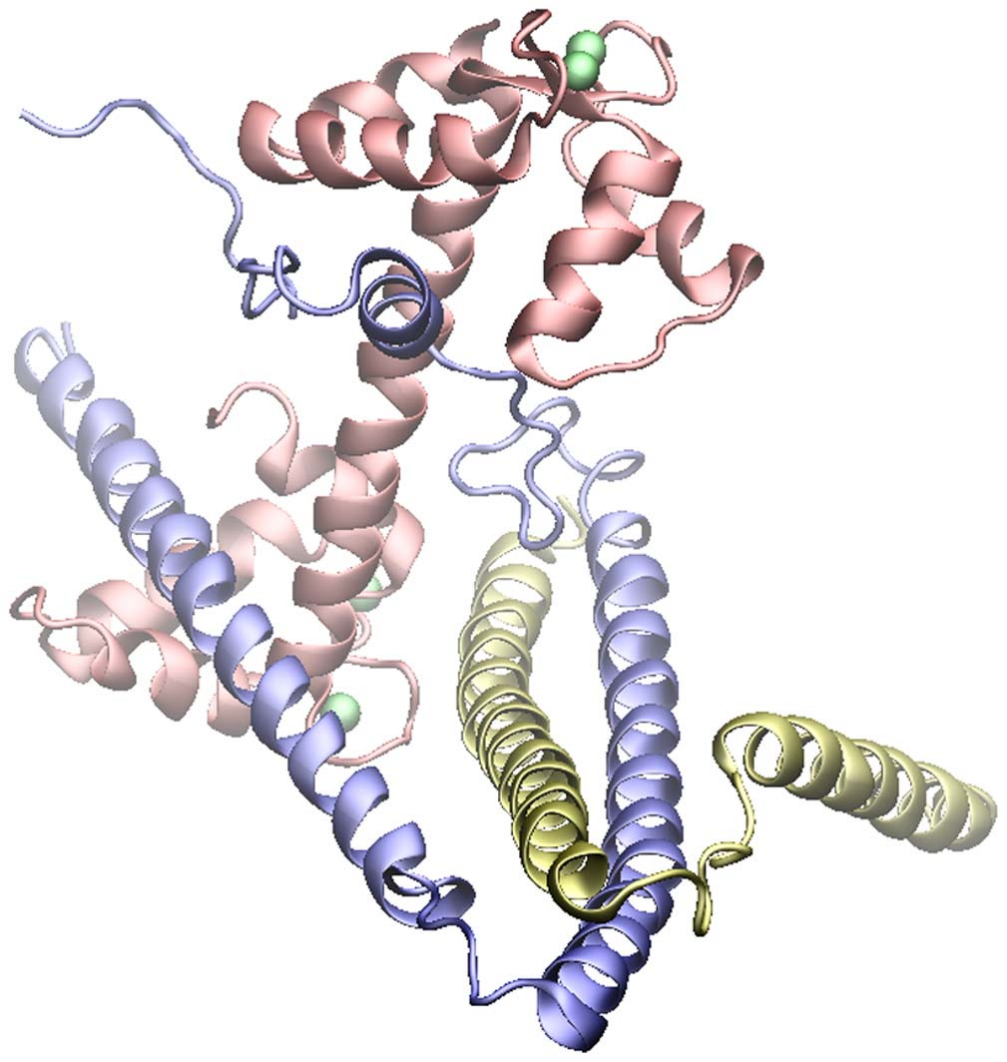
The core domain of the troponin molecule. The Ca^2+^ sensor troponin consist of 3 subunits. The TnI subunit is shown in blue, the TnC subunit is shown in red and the TnT subunit is show in yellow. Ca^2+^ ions located in sites I through IV are shown as green vdW spheres. Sites III and IV located in the IT arm have a higher affinity to Ca^2+^ and do not serve a regulatory role. The N-terminal of TnT, the C-terminal of TnT and the C-terminal of TnI are not present in the crystal structure and are not displayed herein. Molecular coordinates were obtained from 1YTZ.pdb.

The interaction between Ca^2+^ and the regulatory site(s) of striated muscle regulatory protein troponin switches on and off muscle contraction. Troponin is composed of three subunits; troponin C (the Ca^2+^-binding subunit), troponin I (the inhibitory subunit) and troponin T (the tropomyosin-binding subunit) (Figure 1). Ca^2+^ binding to the regulatory site(s) of TnC triggers a series of conformational changes in the troponin (Tn) complex, allows tropomyosin to move along the actin filaments and allows actin to interact with myosin to produce force [1].

TnC is a member of the EF-hand Ca^2+^-binding proteins with four Ca^2+^-binding sites. TnC consists of two globular lobes, the N- and the C-lobe, connected with a central linker. Each lobe contains a pair of EF-hand Ca^2+^-binding sites. The Ca^2+^-binding sites in the N-lobe are believed to be responsible for the regulation of muscle contraction, whereas those in the C-lobe show higher affinities for Ca^2+^ and play a structural role. In cardiac muscle, TnC has only one functional regulatory Ca^2+^-binding site (site II) in the N-lobe due to the replacement of the key Ca^2+^-coordinating amino acid residues in site I [2]. Unlike calmodulin, which is another example of the EF-hand Ca^2+^-binding proteins with four Ca^2+^-binding sites and recognizes a number of target proteins, TnC is known to have only one Ca^2+^-dependent target protein - TnI. The switch region of TnI interacts with the N-lobe of TnC in a Ca^2+^-dependent manner.

TnC has been a target for therapeutics. Drugs that affect TnC’s affinity for Ca^2+^ directly or indirectly have been developed to treat patients with heart failure or with neuromuscular diseases [3], 4. Understanding of structural changes upon Ca^2+^ binding to, or Ca^2+^release from TnC should help the effective development of such drugs.

Molecular dynamics (MD) simulations are a suitable and effective approach to delineate key events during activation and relaxation at an atomic level. MD simulations explore the atomic level details of molecular motion of macromolecules by means of computing the Newtonian equations of motion for each atom to obtain the time dependent evolution of the molecular system. Such simulations, starting with the groundbreaking work on BPTI in the late 1970s [5], have revealed the source of mechanical strength of the sarcomeric protein titin [6], the folding of the BPTI molecule on millisecond time scale, the folding of the WW domain [7], [8] and the dynamics of the STM virus in a system of over 1 million atoms [9], to name a few.

EF-hand proteins have been the subject of several computational studies in recent years [10], [11], [12] and so has been another thin filament protein - actin. Deriu et al. [13], [14] used atomistic and continuum scale modeling to describe the macro mechanics of actin filaments as a function of nanoscale events on the local level using molecular dynamics, coarse-grained model and normal mode analysis. Fan et al. [15] focused on the structural polymorphism of the actin filament and explored the heterogeneity in, and interactions between, the dynamic domains of the filament using coarse grained analysis.

Molecular dynamics has also been used in the study of the troponin complex. Recent works analyzed the correlated motions in the Tn subunits and Tn inter domain interactions [16] and identified and designed putative ligands that can bind and fit into the hydrophobic pocket of the TnC N-lobe [17]. In an earlier interdisciplinary study, Ertz-Berger et al. [18] used molecular dynamics of the TnT domain (residues 70–170) to first explore the effect of mutations and then validate the computational predictions using transgenic mice. The well known codon Arg-92, a mutational hotspot, was mutated in silico to Trp and Leu and effects of the mutations in cTnT on the molecular flexibility of cTnT were studied with MD. The opening motion of the N-lobe of cardiac TnC was explored in a multi scale computational study by Lindert et al. [19]. Ca^2+^ binding to Site I of cardiac Tn was observed to induce correlative motions in the EF-hand and domain movements that precipitate the forming of the TnI interface.

Troponin binds Ca^2+^ at sites I and II in the N-lobe of TnC. Site I is a Ca^2+^-binding loop formed by TnC residues 29–40 and site II is a Ca^2+^-binding loop formed by TnC residues 65 to 76 (chicken fast skeletal isoform residue numbers). The key function of the EF-hand regulatory domain is to sense a Ca^2+^ chemical signal and undergo a conformational change which enables the further downstream transduction of the signal. In the case of TnC it has long been considered that this conformational change is a closure of the N-lobe A/B helix hydrophobic pocket and that in the absence of Ca^2+^ at the regulatory sites the N-lobe of TnC forms a “closed” structure; then upon Ca^2+^-binding to the regulatory sites, it adopts a “open” structure to provide TnI-interaction sites. Although these “final” structures have been investigated relatively well, the pathway between these two structures has not been understood. Additionally it has been proposed [20] that in the process of thin filament relaxation, upon Ca^2+^ release from the TnC sites I and II, the TnI switch region is expelled from the TnC binding pocket which then closes. Upon Ca^2+^ binding, the pocket opens and the TnI switch region is re-inserted into the pocket. Despite the rich body of experimental and computational evidence unanswered questions regarding whether the EF-hand motif in general, and the TnC N-lobe in particular, is a bi-stable switch shuttling between open and closed states remain.

Indeed, blockage of the opening motion of the N-lobe by intra-domain disulphide bridging results in the practical disappearance of the Ca^2+^-dependent regulatory function of Tn [21]. The first TnC structural study [22] presented a Ca^2+^-depleted N-lobe with a fully closed hydrophobic pocket and in subsequent work [23] the authors postulated the HMJ model conceptualizing that binding of Ca^2+^ causes the opening of the hydrophobic pocket for further interaction with TnI. In the case of cardiac TnC however the hydrophobic pocket of the N-lobe maintains a closed conformation after Ca^2+^ binds to site II while Site I does not bind Ca^2+^ at all, as was shown in several structural studies [24], [25], [26]. Small molecule binding (bepridil) or protein ligand (TnI) causes the hydrophobic pocket to open [27], [28]. Computational and experimental studies of calmodulin have shown a fluctuation of the N-lobe between open and closed states [29] or a lesser opening of the N-lobe [30] and further emphasized the backbone plasticity of the EF-hand proteins [31]. Grabarek’s EF β-scaffold model suggests that subtle changes in the backbone dihedral φ, ψ angles in the N-lobe β-sheet allow the N-lobe to sample different conformations until a target specific one is reached.

In this study, we used MD simulations to characterize the Ca^2+^-dependent dynamics of the fast skeletal troponin molecule and the TnC subunit in the Ca^2+^-saturated and depleted states. We focused on the N-lobe and on describing the atomic level events that take place subsequent to removal of the Ca^2+^ ion from the regulatory sites I and II. This approach allowed us not only to compare the initial and final states of troponin but to also explore the dynamics of troponin in the activated and deactivated state; to explore the dynamics that take place subsequent to Ca^2+^ release; to reveal the atomic level details of the conformational changes that occur in response to Ca^2+^ release; and to characterize the transitional events leading to thin filament relaxation.

## Materials and Methods

Molecular dynamic simulations were performed using the NAMD [32] software package running on the XSEDE Lonestar supercomputer. The crystal structure files of fully Ca^2+^-saturated avian fast skeletal troponin molecule (1YTZ.pdb) [20] and Ca^2+^-depleted TnC subunit (5TnC.pdb) [33] were obtained from the Protein Data Bank [34].

Our choice of PDB system was driven by the considerations that while both Ca^2+^- and apo-form crystal structures are available for the core domain of the skeletal troponin complex, 1YTZ.pdb is the only available structure in the Ca^2+^ form. The 5TnC.pdb crystal structure of Herzberg and James is the first crystal structure of EF-hand type Ca^2+^-binding proteins, is determined at 2 Å resolution, and is widely considered to be the gold standard for skeletal TnC structural description. The closeness of the two source species: Gallus gallus (1YTZ.pdb) and Meleagris gallopavo (5TnC.pdb) provided an additional level of complementarity. It should be noted that the selected PDB files have some missing residues. Specifically, in 1YTZ.pdb the 39 C-terminal amino acid residues (144–182) of TnI or the C-terminal residues of TnT2 (249–262), as well as the N-terminal tail domain of TnT, are not in the crystal set. We did not incorporate these residues in our simulations. These residues are considered to be flexible, at least in the absence of tropomyosin and it was demonstrated that the C-terminal mobile domain of TnI behaves independently from the core domain of the complex by NMR measurements [35], [36].

In total we prepared and simulated 8 systems: 1) core troponin (TnI, TnC, TnT), Ca^2+^ present in sites I, II, III and IV (Tn_4_Ca^2+^); 2) core troponin (TnI, TnC, TnT), Ca^2+^ present in sites III and IV only (Tn_2_Ca^2+^); 3) TnC domain, Ca^2+^ present in sites I, II, III and IV (TnC_4_Ca^2+^); 4) TnC domain, Ca^2+^ present in sites III and IV (TnC_2_Ca^2+^); 5) TnC domain where site I was depleted, site II was saturated (TnCSite1noCa^2+^); 6) TnC domain, where site I was saturated, site II was depleted (TnCSite2noCa^2+^); 7) TnC domain, Ca^2+^ present in sites III and IV, with residue ALA24 mutated to ASP24 and LEU48 to GLU48 (TnCASP24GLU48), and 8) TnC domain, Ca^2+^ present in Site III and IV (5TnC). Protein initial coordinates for systems 1 through 7 were derived from 1YTZ.pdb and protein initial coordinates for system 8 were derived from 5TnC.pdb. Protein structure files (psf) were created using the molecular modeling package VMD [37] and the plug-in program psfgen. Hydrogen atoms were added and the protein systems were solvated in explicit solvent environment. The CHARMM [38] force field was used for the protein, water was considered as the TIP3P model [39]. The total system size for the molecular structures ranged from 55582 to 87325 atoms. The molecular systems were energy minimized by conjugate gradient method and heated to 300 K. Simulations were performed with periodic boundary conditions in the NPT ensemble; temperature and pressure were kept constant by employing Langevin dynamics, at 1 atm and 300 K. Electrostatic interactions were computed by PME (Particle mesh Ewald) method. Non bonded interactions were treated with a cutoff using a switching function beginning at 10 Å and reaching zero at 14 Å. Data extraction, trajectory analysis, figures and visualizations were completed using VMD and MATLAB. In total, 16 simulations were run and 0.48 µs of simulation time were collected and analyzed. Table S1 presents a list of systems, number and duration of simulations performed.

## Results and Discussion

In this work we explored via molecular dynamics simulations the dynamics of the calcium sensor protein troponin and the events occurring after Ca^2+^ release from the TnC N-lobe regulatory binding sites. A closure of the hydrophobic pocket results from the integrated effect of the following conformational events: breakage of H-bond interactions between the backbone nitrogen atoms of the residues at positions 2, 9 and the sidechain oxygen atoms of the residue at position 12 (N^2^-OE^12^/N^9^-OE^12^) in sites I and II; expansion of sites I and II and increased site II N-terminal end-segment flexibility; strengthening of the β-sheet scaffold and the subsequent hydrophobic re-packing of the N-lobe hydrophobic residues. Additionally the Ca^2+^ release allows the N-lobe to rotate 25–35 degrees relative to the rest of the Tn molecule.

We performed equilibrium molecular dynamics simulations on the TnC subunit (residues 3 to 161) and the core domain of the troponin complex. The following set of systems were examined: first, open TnC systems where Ca^2+^ was removed from sites I and II, only from site I, only from site II and not removed at all (molecular coordinates derived from Ca^2+^-saturated structure - 1YTZ.pdb); second, the closed, deactivated TnC domain (molecular coordinates derived from the Ca^2+^-depleted TnC - 5TnC.pdb); and third, the troponin molecule Ca^2+^-saturated and Ca^2+^-depleted state (molecular coordinates derived from 1YTZ.pdb). Thus, we simulated the open activated skeletal Tn and TnC domain in the Ca^2+^-saturated state; and then to model the structural events leading to thin filament relaxation, we removed Ca^2+^ from sites I and II of open TnC and simulated the resulting structure. To access the conformational coupling between the two sites and the contribution of each site to the structural transitions of the N-lobe we also simulated structures with only one site occupied. The 5TnC based system served to establish a frame of reference to which to compare the structural transitions discerned from the simulations.

### Dynamics of the N-lobe and Closing of the A/B Helix Hydrophobic Pocket

The level of openness of the TnC N-lobe hydrophobic pocket in the holo and apo state and the closing of the pocket can be observed and measured in a molecular dynamics simulation trajectory. To establish a consistent metric across all our simulations we measured the openness of the pocket as the distance *d* between the alpha-C atoms of the first residue of the A helix (GLU16) and the last residue of the B helix (LEU48). For the closed 5TnC system the pocket openness distance was d = 13.65 Å±0.71 (averaged over the duration of the simulation). For the open, Ca^2+^-depleted TnC system the pocket rapidly closed to d = 13.23 Å±0.60 (averaged over the last 10 ns of the 3 TnC_2_Ca^2+^ simulations). The open, Ca^2+^-saturated systems TnC pocket closed to d = 15.74 Å±1.29 (averaged over the last 10 ns of the 3 TnC_4_Ca^2+^ simulations). The open, site I-depleted, site II-saturated TnC system (TnCSite1noCa^2+^) closed to d = 16.48 Å±0.86 (averaged over the last 10 ns of the simulation), whereas the open site I-saturated, site II-depleted TnC system (TnCSite2noCa^2+^) closed to d = 15.18 Å±1.10 (averaged over the last 10 ns of the simulation). In the presence of TnI, the TnC pocket did not close in either Ca^2+^-saturated or depleted systems for the duration of the MD simulations and remained at d = 23.87 Å±0.89 and d = 23.79±0.94 Å respectively (averaged over the duration of the 3 Tn_4_Ca^2+^ and the 3 Tn_2_Ca^2+^ simulations).

**Figure 2:**
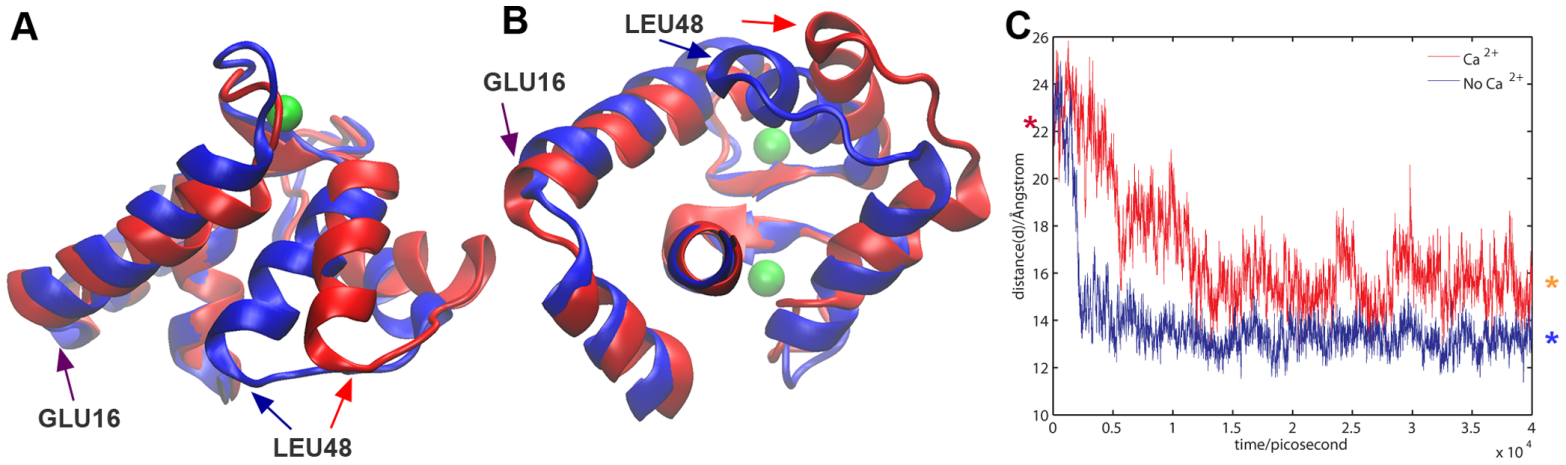
Open and closed state of the N-lobe. Time dependence of TnC hydrophobic pocket opening distance (d). **A, B** The TnC N-lobe is shown in red in the Ca^2+^ saturated, open state; and in blue in the Ca^2+^ depleted, closed state. Panel A shows the N-lobe viewed from the side, panel B shows the N-lobe viewed from the C-lobe. The open and closed state structures are aligned along the D helix. Ca^2+^ ions are shown as green vdW spheres and TnC residues with resid>85 are not shown. **C.** Openness of the TnC N lobe A/B helix hydrophobic pocket measured as the distance *d* between alpha-C of GLU16 and alpha-C of LEU48 in saturated TnC and depleted TnC. At the start of the simulation both TnC structures (depleted and saturated) are in the open state (marked by purple asterisk) and evolve to a closed state (marked by light blue asterisk) for the depleted TnC or semi-closed state (marked by orange asterisk) for the saturated TnC. The blue line depicts the time dependent evolution of the pocket opening distance *d* of depleted TnC; the red line depicts the time dependent evolution of the pocket opening distance *d* of saturated TnC.

Thus, the N-lobe of TnC in the absence of TnI did not remain open but reached a semi-closed state if the regulatory sites were occupied by Ca^2+^ and reached a closed state only if the regulatory sites were unoccupied (Figure 2). Thus a “semi-closed” conformation of the EF N-lobe may present a more probable state and the open conformation that is observed in structural work could stem from stabilization effects of the crystal lattice as suggested in [29].

### Root Mean Square Fluctuations of the N-lobe

**Figure 3:**
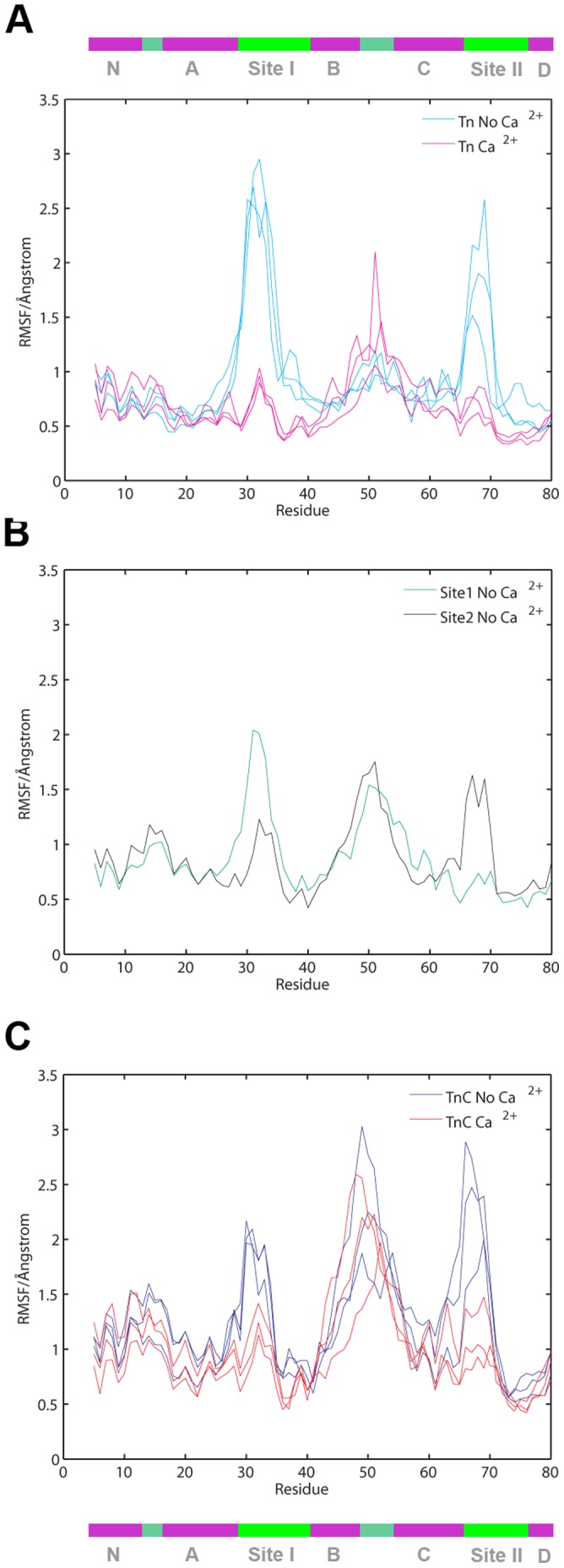
RMSF of TnC N-lobe. A. RMSF for each residue alpha-C of TnC N-lobe (residues 5–80) measured in the core troponin complex (TnC-TnT-TnI) simulations. Ca^2+^ depleted observation shown in cyan, Ca^2+^ saturated observation shown in magenta. B. RMSF for each residue alpha-C of TnC N-lobe (residues 5–80) measured in site occupied/site depleted simulations. Measurement for TnCSite1NoCa^2+^ shown in green and TnC Site2NoCa^2+^ shown in black. C. RMSF for each residue alpha-C of TnC N-lobe (residues 5–80) measured in TnC simulations. Ca^2+^ depleted observation shown in blue, Ca^2+^ saturated observation shown in red. Colorful bar in the top and bottom of the figure shows the segments of the N-lobe as related to residue number.

The root mean square fluctuations (RMSF) of TnC N-lobe residues (residues 5 to 80) were measured and are presented on Figure 3. The regions of interest where fluctuations are Ca^2+^ state correlated are: first region - corresponding to Ca^2+^-binding site I; second region - corresponding to the B helix, linker and C helix segment; and third region - corresponding to Ca^2+^-binding site II. The regions have been coded as a colorful band in the upper segment of Figure 3.

In the simulation of the core troponin complex (Tn_4_Ca^2+^) in Ca^2+^-saturated state the observed RMSF at the saturated Ca^2+^-binding sites does not exceed 1Å (Figure 3A); similarly in the TnC domain simulations the observed RMSF at the saturated Ca^2+^-binding sites does not exceed 1.5 Å (Figure 3B, C). Release of Ca^ 2+^ induces up to 3-fold increase of RMSF at the depleted Ca^2+^-binding site, with the most significant jump centered at the N-terminal segment of each unoccupied site. Comparable behavior was observed in both the troponin complex and TnC only simulations. In the two TnC simulation where one site was occupied while the other one was depleted (TnCSite1noCa^2+^ and TnCSite2noCa^2+^), the depleted site shows significant increase of fluctuations vis-à-vis the saturated site. Additionally in the TnC simulations another RMSF peak was observed centered at residue GLN50. Thus the level of RMSF of each binding site depends on its Ca^2+^-state and the RMSF peak present in the B/C connected loop region is accounted by the movement of B/C helices towards the D helix which precipitates the closure of the A/B hydrophobic pocket observed in the TnC simulations.(Figure 3B,C). Additionally, presence of TnI further stabilizes the N-lobe regions.

### Structural Characterization of the Open to Closed Transition in Skeletal TnC

The thin filament relaxation pathway commences with Ca^2+^ release from TnC N-lobe sites I and II. We modeled this process by removing Ca^2+^ from site I and II of the open, saturated TnC structure and commencing our simulations from this starting point which would correspond to the moment subsequent to Ca^2+^ release from the troponin switch in vivo. The closure of the A/B helix hydrophobic pocket is caused by the occurrence and integrated effect of the following events which we now describe.

#### 1. Breakage of the N^2^-OE^12^/N^9^-OE^12^ interactions, expansion of the Ca^2+^-binding sites

The Ca^2+^ ion is coordinated by the residues at positions 1, 3, (5 in site II), 7 and 12 of Ca^2+^-binding loop at the two sites [40], [41]. The 12^th^ position bidentate ligand is a highly invariant GLU residue. Particularly important for the regulatory function of the troponin switch is a set of interactions that occur between the Ca^2+^ ion, sidechain OE1 and OE2 of GLU^12^ (residues GLU40 and GLU76) and the backbone N atoms of the residues at positions 2 and 9 (ALA30/GLU66 and SER37/ASP73) (denoted here as N^2^-OE^12^/N^9^-OE^12^). Thus in the Ca^2+^-bound state the sidechain oxygen atoms of the GLU^12^ are held in towards the Ca^2+^-binding pocket by a bi-dentate charge-charge interaction to the Ca^2+^ ion and by H-bonds towards the main chain nitrogen atoms of the two loop residues.

The importance of this interaction for the regulatory function of the EF-hand motif in calmodulin has been underlined by mutational studies where the GLU was replaced by GLN [42]–[43]. Additionally, NMR work [44] and CD analysis [45] has shown that mutation of the GLU to ALA causes the N-lobe of the TnC subunit to remain in closed-like state and impairs the functionality of the protein even if Ca^2+^ is present in high concentration.

The N^2^-OE^12^/N^9^-OE^12^ interaction is stable and is not broken during our simulation trajectories whenever the binding site is occupied by Ca^2+^. The CD atom of the GLU^12^ side chain in both site I and II maintain a distance to the N^2^ and N^9^ at under 3 Å in the simulation trajectories of Ca^2+^-saturated Tn and Ca^2+^-saturated TnC. In the TnCSite1noCa^2+^ and TnCSite2noCa^2+^ simulations this distance also remains under 3 Å at the Ca^2+^-occupied binding site. The distance between the GLU^12^ OE and the N^2^ is the lesser of the two distances in all cases. Ca^2+^ presence in the binding site is necessary to maintain the N^2^-OE^12^/N^9^-OE^12^ interaction and the motions of the tops of the site- entering and exiting helix are coupled by the ion present in the site.

**Figure 4:**
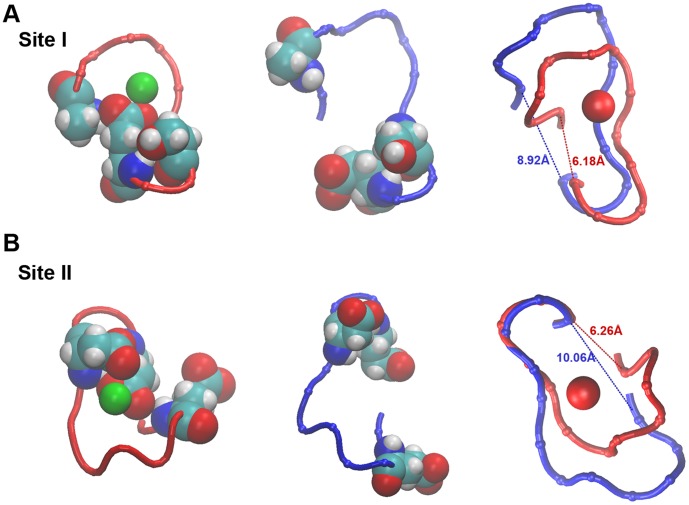
Breakage of the N^2^-OE^12^ and N^9^-OE^12^ ‘prong’ interaction and expansion of the Ca^2+^ binding pocket at sites I and II. Trajectory snapshots for the open and closed state. **A.** Ca^2+^ binding site I is shown in the TnC saturated, open state (left panel) and the depleted, closed state (middle panel). The residues forming the interactions N^2^-OE^12^ and N^9^-OE^12^ are shown as vdW representation (residues ASP30, SER37, GLU40 in site I). The interactions are well maintained in the saturated, open state (red tube) and are released in the depleted, closed state (blue tube). The right panel show a superimposition of site I in the two states. The distance between the alpha-C atoms of the residues located at positions [1], is increased following Ca^2+^ release and the binding pocket expands. **B.** Ca^2+^ binding site II is shown in the TnC saturated, open state (left panel) and the depleted, closed state (middle panel). The residues forming the interactions N^2^-OE^12^ and N^9^-OE^12^ are shown as vdW representation (residues GLU66, ASP73 and GLU76 in site II). The interactions are well maintained in the saturated, open state (red tube) and are released in the depleted, closed state (blue tube). The right panel show a superimposition of site II in the two states. The distance between the alpha-C atoms of the residues located at positions [1], [12] is increased following Ca^2+^ release and the binding pocket expands.

Ca^2+^ release from both binding sites is followed by the immediate breakage and disbanding of the N^2^-OE^12^/N^9^-OE^12^ H-bond interactions (Figure 4). This enhances the motional degrees of freedom at the N-terminal end-segment of the Ca^2+^-binding loop and uncouples it from the C-terminal end-segment which allows position 1 and 12 to separate from each other. At site I, the GLU40^12^ ligands side chain moves away from the Ca^2+^-binding site and the tops of helix A and B become uncoupled. At site II, the GLU76^12^ ligand side chain moves away from the Ca^2+^-binding site and the tops of helix C and D are uncoupled analogously. The combined conformational change after release of Ca^2+^ is a subtle expansion of the Ca^2+-^binding pocket (Figure 4). Notable is that the N^9^-CD^12^ distance remains shorter that then N^2^-CD^12^ distance and the N^9^-OE^12^ H-bond partners remain closer and the bond sometimes reforms.

#### 2. Flexibility of the N-terminal end segment site II

At the same time of the N^2^-OE^12^/N^9^-OE^12^ H-bonds breakage at Site II the side chains of ligands 1, 3, and 5, now free from the interaction with the ion rotate away from the former location of the ion. Thus the entire ASP65-SER69 segment rotates outwards towards the solvent. As helices C and B are coupled to the ASP65-SER69 segment, the lifting motion is propagated down the C helix and allows for the movement of the B and C helices towards the A and D helices. We did not observe any of the N^2^-OE^12^/N^9^-OE^12^ breakage and Site II ligands 1,3 and 5 rotation in the Ca^2+^-saturated TnC simulations (TnC_4_Ca^2+^). In contrast the TnCSite1noCa^2+^ simulation showed the similar N^2^-OE^12^/N^9^-OE^12^ breakage events at site I, however since site II is occupied by the ion, its ligands 1, 3 and 5 are closely held and the N-terminal segment of site II is less flexible. Hence we did not observe a closure of the pocket to the lowest *d* but only to an intermediate value. The opposite was observed in the TnCSite2noCa^2+^ simulation and since in this simulation the site II N-terminal end-segment was more flexible, the hydrophobic pocket was able to close tighter in our TnCSite2noCa^2+^ TnC simulation vis-à-vis the TnCSite1noCa^2+^ TnC simulation.

#### 3. Hydrophobic rearrangement

**Figure 5:**
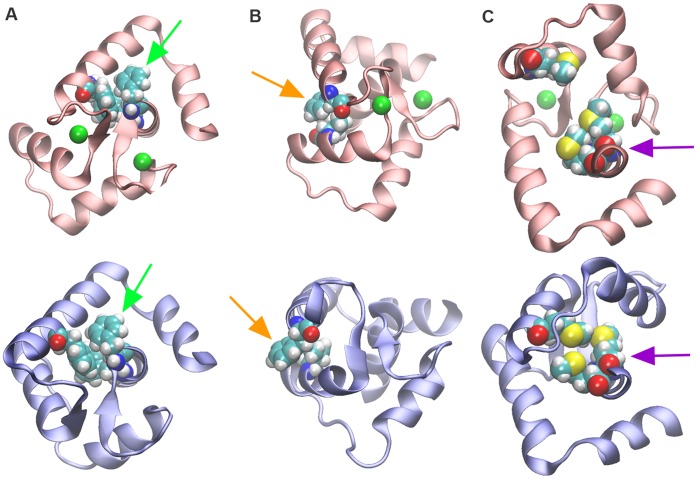
Hydrophobic core and hydrophobic residue re-packing following release of Ca^2+^. In all panels the saturated, open state N-lobe TnC is shown in red and the depleted, closed state N-lobe TnC is shown in blue. **A.** A hydrophobic PHE scaffold remains undisturbed in the open to closed transition. Residues PHE74, PHE77 and PHE25 are shown as vdW spheres. Top panel shows the TnC open state, bottom panel shows the TnC closed state. Green arrows point to PHE74 in each panel. **B.** Expulsion of residue PHE28 into the solvent and sliding of VAL44 into the hydrophobic core in the open to closed transition. Top panel shows the TnC open state, bottom panel shows the TnC closed state. Orange arrows point to PHE28 in each panel. **C.** Tight repacking of MET81, MET80 and MET45, which finish in a tight formation upon closure of the hydrophobic pocket, takes place in the open to closed transition. Top panel shows the TnC open state, bottom panel shows the TnC closed state. Violet arrows point to MET80 and MET81 in each panel.

The N-lobe of TnC is rich in hydrophobic residues. Additionally the N-lobe exhibits a very stable hydrophobic core segment consisting of three PHE residues - PHE25, PHE74 and PHE77. The contact between those 3 residues changed negligibly during all simulations and in the open to closed transition. Therefore the residues serve as a hydrophobic scaffold around which the movement of other residues during closure is coordinated (Figure 5A, Movie S1). The release of the N^2^-OE^12^/N^9^-OE^12^ interaction is followed by a dramatic hydrophobic rearrangement and repacking, which occurs at the A/B hydrophobic pocket. Residue PHE28’s side chain is previously hidden in the hydrophobic core of the protein and in contact with PHE77, LEU41 and VAL44. The increased flexibility in N-terminal end-segment of site I allows the PHE28 aromatic ring to rise up and out and emerge now on the outside of the pocket (Figure 5B, Movie S2). The potential inability of the PHE28 to rise out of the hydrophobic core has a negative effect to the Ca^2+^ sensor function of Tn. An earlier study by Chandra et al. [46] showed that a substitution of the PHE for a bulkier TRP is deleterious. The larger side chain of TRP conceivably would not be able to exit the hydrophobic core and position above the VAL44 thus negatively impacting the ability of the hydrophobic pocket to contract. The rise of PHE28 was also observed in computational work by Depuis at al [12] with a different simulation method.

VAL44, which starts in a very close contact with PHE28, moves into and then past the space previously occupied by (and now vacated by the rotation of) the PHE28’s side chain and then inserts deep into the hydrophobic core of the N-lobe by making a contact with PHE77, PHE25 (the core scaffold phenylalanines) and ALA24. The side chain of the VAL44 finishes positioned under the aromatic ring of the PHE28 and into PHE77.

The two opposing methionines MET27 and MET47 change the relative position of their side chains and MET27’s side chain moves towards and slides under the side chain of MET47. The flexible S-CH_3_ ends of their side chains orient toward one another and seal the hydrophobic packing of the A/B pocket from below. THR43 rotates its side chain so that the hydroxyl group is towards the solvent and the methylene group is towards the inside of the pocket where it will meet the rising side chain of the PHE28.

Additional conformational changes and hydrophobic rearrangement occurs resulting in a tighter hydrophobic packing between ILE60, ILE61, VAL64 on the C helix, ILE72 in the site II loop and PHE77, MET80 and MET81. MET80 side chain slides under ILE60 and packs towards LEU41 on the B helix. MET81 adjusts its flexible end to form a contact with LEU48 since the two residues end up close to each other as a result of the closing motion of the B/C helices. The three methionines MET81, MET45 and MET80 meet and the side chain of the MET45 inserts in the groove between the other two MET residues of the D helix, with the flexible end segments of all three residues sampling different rotamers until a tight packing fit is reached (Figure 5C, Movie S3).

#### 4. Strengthening of the β-sheet scaffold

**Figure 6:**
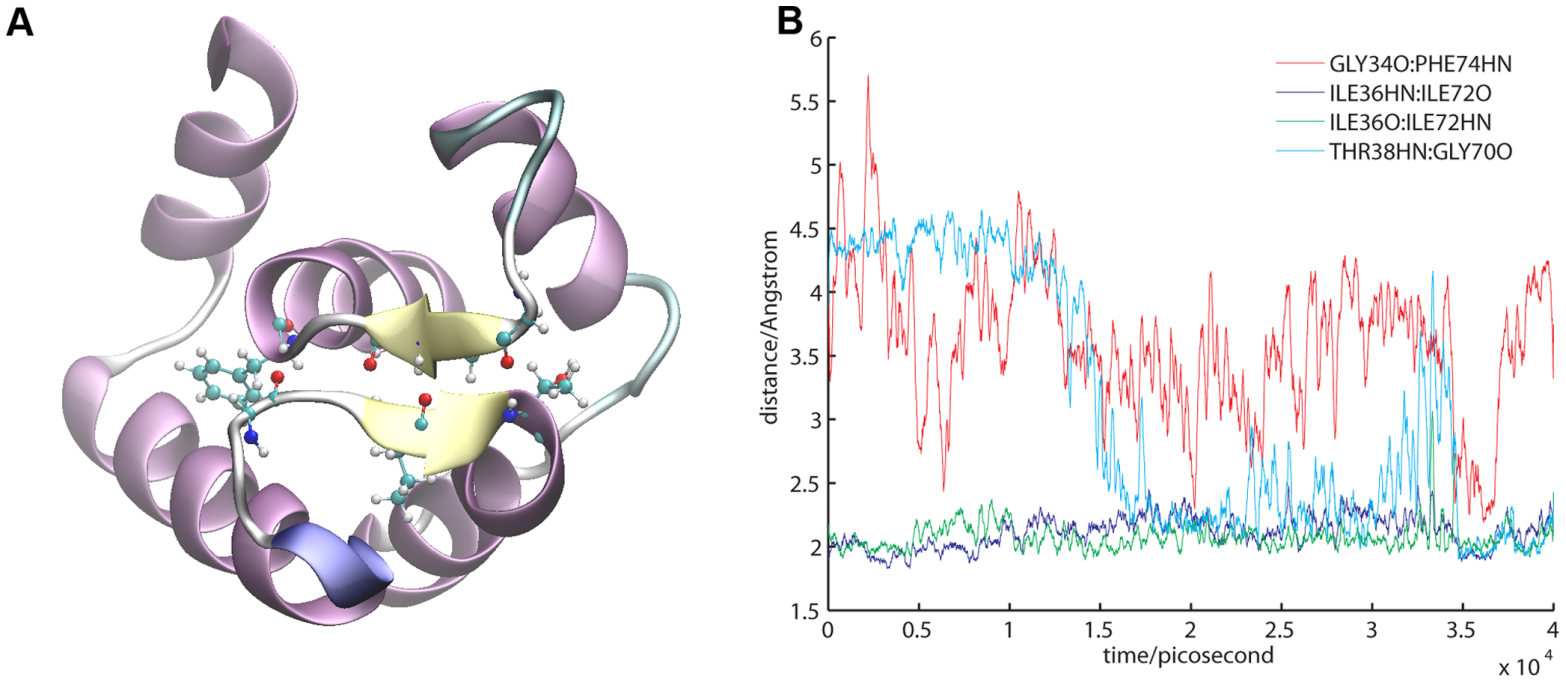
Consolidation of the β-sheet upon Ca^2+^ release. A. Structural view of the small β-sheet located at the ‘top’ of the TnC N-lobe. Key residues GLY34, ILE36, THR38, GLY70, ILE 72 and PHE74 shows as CPK representation. **B.** Distances between HN and acceptor O for each of the four H-bond partners are shown. The time evolution of the distances is measured in the open to closed transition in a simulation of Ca^2+^ depleted TnC.

The motions of the two Ca^2+^-binding sites are coupled via the β-sheet structure located on top of the N-lobe (Figure 6A). The two central bonds acceptor-donor pairs are formed by the main chain oxygen and nitrogen atoms of ILE36 and ILE72 while GLY34-PHE74 and THR38-GLY70 are also possible H-bond partners. The importance of this structure has been deeply discussed by Grabarek [47], [48] and his work gives rise to the EF β-scaffold model of Ca^2+^ regulation of EF-hand featuring proteins. The EF β-scaffold model interprets the Ca^2+^ binding mechanism as a two step process where the binding of the ion as well as the subsequent conformational re-arrangements are driven by the β-sheet structure. The model couples the changes in the backbone dihedrals of the two opposing ILE residues 36 and 72 to the conformational events that lead to the movement of the BC helices and the opening of the pocket. The same recent computational study [12] utilizing ART nouveau sampling [49] observed and interpreted consolidation events in the EF β-scaffold in support of this model.

In the holo state troponin complex and TnC simulations we observed steady hydrogen bonding between ILE36O-ILE72N and ILE36N-ILE72O while the donor-acceptor distance between GLY34-PHE74 and THR38-GLY70 remained well over 4 Å. Ca^2+^ release from both site I and site II induces a change in the dynamics of the β-sheet (Figure 6B). The THR38HN-GLY70O distance collapsed to under 2.5 Å. Notable here is that this H-bond strengthened even in the presence of TnI. The GLY34O-PHE74HN distance showed the greatest fluctuation: it ranged between 2 Å and 8 Å in the simulations of the Ca^2+^-depleted state. The distances observed for the four H-bonding partners compared well to the distances measured in the apo TnC simulation (5TnC).

### Semi-closed State of TnC N-lobe

Ca^2+^-saturated or semi-saturated TnC reaches a semi-closed conformation in the absence of TnI. In this state the B/C helices move towards the A/D helices, the hydrophobic pocket opening is tightened and the opening distance reduced. The saturated TnC N-lobe however is not fully closed to the degree observed in the simulations of Ca^2+^-depleted state. The semi-closed state is a conformation resulting from the partial closure of the N-lobe within the constraints imposed by the Ca^2+^ ion present in the site I and II Ca^2+^-binding pockets (Figure S1).

Structural comparison of the open and semi-closed state reveals that the N^2^-OE^12^/N^9^-OE^12^ interactions at site I and site II are present and well maintained throughout the trajectories and the two sidechain oxygen atoms of GLU40 and GLU76 are firmly pointed towards the Ca^2+^ ion and towards the nitrogen atoms at position 2 and 9. There is also little difference in the packing of the core PHE25, PHE74 and PHE77. The packing of other N-lobe hydrophobic residues however show remarkable differences between the open, semi-closed and closed states. The PHE28 side chain reaches out to a lesser degree in the semi-closed state while VAL44 is able to approach PHE77 less. Since the N^2^-OE^12^/N^9^-OE^12^ interaction at sites I and II and the Ca^2+^ ion are still present, the PHE28 side chain cannot escape further out and only a semi-closed state can be reached. In the closed state the aromatic ring of PHE28 is positioned above the B helix and over the main chain nitrogen atom of VAL44 whereas in the open and semi-closed state the aromatic ring is further back into the space between the A and B helices. The VAL44 side chain is thus either in contact with the aromatic ring of PHE28 or pointed towards the aromatic ring of PHE77 and PHE25. The packing of the hydrophobic residues between the D helix and the B helix is also less complete - the three methionines: MET81(D), MET45(B) and MET80(D) are further separated in the semi-closed than in the closed state.

Additionally, the THR38N-GLY70O H-bond at the β sheet in the closed state is well formed at a distance of approximately 2 Å whereas in the semi-closed and open state, the distance is stable at over 4.7 Å, except in the case of the TnCSite2noCa^2+^ simulation where the release of the ion allows the formation of the THR38N-GLY70O H-bond.

In our simulations the TnC subunit collapsed to a semi-closed state accessible in the absence of TnI and the presence of Ca^2+^. The existence of this state brings evidence to the innate ability of the TnC N-lobe to undergo a partial closure even when the trigger Ca^2+^ ion is present. The key difference however is that the removal of Ca^2+^ breaks the N^2^-OE^12^/N^9^-OE^12^ ‘prong’ interaction; liberates the N-terminal end segment of the binding site II thus allowing the B helix VAL44 to slide swiftly under the rising aromatic ring of the A helix’s PHE28 and make a contact with the PHE77 of the unperturbed hydrophobic core scaffold (PHE77, PHE74 and PHE25) of the N-lobe.

### N-lobe Sensitivity to Mutations

Closure of the hydrophobic pocket is critical in the discharge of the Ca^2+^ modulated regulatory function of troponin. The closure motion would likely be very sensitive to residue mutations that would disrupt the hydrophobic fabric of the pocket. To test this notion as to prevent the closing of the TnC hydrophobic pocket when the Ca^2+^ ion is removed we mutated two hydrophobic residues into charged residues. Residue ALA24 and residue LEU48 were mutated into hydrophilic residues - ASP(24) and GLU(48) (Figure S2 A). The mutated TnC was then subjected to equilibrium MD for 5 nanoseconds and the dynamics behavior was compared to that of the WT protein. In the simulation of ASP24GLU48 TnC no decrease in pocket opening distance was observed - we measured opening distance d = 23.75 Å±0.68 (averaged over the duration of the simulation) (Figure S2 B). The suggested mutation is likely to prevent the A/B hydrophobic pocket from closing by steric hindrance and repulsive electrostatic interaction.

### Rotation of the N-lobe

Removal of Ca^2+^ from binding sites I and II in the TnC N-terminal domain induces a rotation of the TnC N-terminal domain relative to the rest of the TnC domain and to the IT arm of the troponin molecule. We measured the rotation of the TnC N-terminal segment relative to the C-terminal TnC segment and the rest of the Tn by comparing virtual dihedral angles formed by planes determined by alpha-C atoms suitably selected from different points of the Tn molecule (Figure S3 A, B). The magnitude of the rotation was in the range of 25–35 degrees (Figure S3 C) which is in qualitative agreement with the comparison of the apo and holo state of Tn (38 degrees rotation) reported by Vinogradova et al. [20]. A recent computational paper from Project at al. also observed a rotation of the N-lobe relative to the C lobe in the protein CaM [10]. Although the rotation may not have a regulatory function in CaM, in Tn it may provide a key potential step in the activation-deactivation mechanism. In TnC such conformation change would couple the movement of the TnI inhibitory region and positioning along the actin filament and the Ca^2+^ state the N-lobe regulatory sites.

### A Model of the Structural Events during the Thin Filament Regulation

Based on our findings we propose a model for thin filament regulation. The deactivation of troponin can be interpreted as a closure-rotation conformational change occurring in response to Ca^2+^ release. Subsequent to Ca^2+^ removal from regulatory sites I and II, the N-lobe hydrophobic pocket closes, while the rising PHE28 residue pushes the TnI switch region towards the front of the pocket while the entire N-lobe rotates. The rotation of the TnC causes displacement of the TnI regulatory segment onto actin. TnI is now poised to inhibit the actomyosin cross-bridge cycling. Full release of the TnI switch region from the TnC binding pocket may not be necessary for the deactivation process. Since the A helix is a full turn longer than the B helix, a comfortable binding interface (MET17, PHE21, LEU48) is presented for TnI to remain in close contact with the closed N-lobe pocket. Subsequent events may include melting of the TnC connecting helix as suggested by Vinogradova et al. [20]. Activation of the thin filament will be then precipitated by rotation and a re-opening of the hydrophobic pocket that can be envisioned as a reversal of the above described steps. The Ca^2+^ ligands will reach [11] to grab and pull in the ion. The sidechain oxygen atoms of the site-terminal GLU and ligands 1, 3 and 5 will be attracted to the ion thus rotating the backbone, restoring the N^2^-OE^12^/N^9^-OE^12^ interactions and inducing the B helix to move away from the A helix while unpacking the hydrophobic core. TnI switch region is then readily re-inserted in the opened pocket. Rotation in the opposite direction occurs at the same time. The combined opening-rotation repositions the TnI inhibitory region away from actin and subsequently exposes the actomyosin interaction sites.

### Conclusions

This study focused on the troponin molecule in isolation, as have other recent computational studies of troponin [16], [17], [50]. This fact notwithstanding, a discussion of the importance of protein-protein interaction involving troponin and other thin filament proteins should be considered. From electron microscope images [51], we do know that the troponin complex can be divided into two domains-the tail and core domains. Crystal structures of the core domain are only available to date and PDB entry 1YTZ is one of them. The tail domain is known to interact with tropomyosin [2]. Among the core domain, the inhibitory region and the C-terminal mobile domain of TnI are thought to interact with actin in the apo state [2]. Recent work [52] suggests that the N-terminal helix of TnT in the core domain of troponin and the C-terminal segment of TnT may interact with actin and tropomyosin. Incorporating these observations, there are several models [53], [54], [55] of the actin-tropomyosin-core troponin domain complex. Yet the exact location and orientation of the core domain of the troponin complex along the actin-tropomyosin filaments has been elusive so far. The structural opening of the N-lobe of TnC was investigated in various complexes in cardiac system using FRET [56] and it was found that the structural opening was slightly constrained by the presence of actin (∼1 Å in the distance measurements of the aforementioned study). Yet 1 Å can be attributed to the orientation difference of fluorescence probes. Thus there has been no clear evidence for interactions that involve the N-lobe of TnC. Nevertheless; molecular dynamics investigations of troponin in the presence of other thin filament proteins would further reveal the dynamic behavior of this sensor system and we plan to pursue this direction in the future.

## Supporting Information

Figure S1 **Open, semi-closed and closed states of TnC N-lobe. A.** Structural comparison of the open and semi-closed states of TnC N-lobe. (shown in red and orange respectively). **B**. Structural comparison of the semi-closed and closed state of TnC N-lobe (shown in blue and orange, respectively). The helices of the N-lobe are labeled accordingly and the top panels show a ‘bird’s eye’ view of the N-lobe while the bottom panels show side views. The Ca^2+^ ions are shown as vdW shapes and in red and orange depending on if they are located in the open or semi-closed state structures. There are no Ca^2+^ ions in the closed state structures.
(TIF)

Figure S2 **A mutation to prevent the TnC hydrophobic pocket from closure. A**. Mutation of TnC N-lobe hydrophobic pocket residues ALA24 and LEU48 to ASP and GLU, respectively. The mutated residues are shown in surface representation and are seen protruding into the pocket. **B**. The evolution of the openness of the hydrophobic pocket of TnCASP24GLU48 in the Ca^2+^ depleted state as measured by the distance *d* between the alpha-C atoms of residues GLU16 and GLU48.
(TIF)

Figure S3 **Rotation of the TnC N-lobe. A**: Virtual dihedral 1 is determined by alpha-C atoms of residues GLU131, PHE104, ASP73, GLU15. The intersection of the two planes formed by alpha-C atoms of (GLU131, PHE104, ASP73) and alpha-C atoms of (PHE104, ASP73, GLU15) runs along the α-helix connecting the N-lobe and C-lobe of TnC. Residue 131 is located in the C-lobe of TnC. Residue 104 and 73 form a segment that spans the TnC connecting helix and Residue 15 is located at the start of the A helix. **B**: The virtual dihedral 2 is determined by alpha-C atoms of residues GLU131, PHE104, ASP73, GLU54. The intersection of the two planes formed by alpha-C atoms of (GLU131, PHE104, ASP73) and alpha-C atoms of (PHE104, ASP73, GLU54) runs along the α-helix connecting the N-lobe and C-lobe domains of TnC. Residue 131 is located in the C-terminal domain TnC. Residue 104 and 73 form a segment that spans the TnC connecting helix and Residue 54 is located at the start of the C helix. Alpha-C atoms of residues GLU131, PHE104, ASP73, GLU75, GLU15 and GLU54 of TnC shown as green vdW spheres. **C**. (**Left panel**) Time dependency of Virtual dihedral 1. Red line depicts the Tn_4_Ca^2+^ structure and blue line depicts theTn_2_Ca^2+^ structure. Tn_2_Ca^2+^ simulation was started from the equilibrated Tn_4_Ca^2+^ structure at 8 nanoseconds (marked with *). **C: (Right panel)** Time dependency of Virtual dihedral 2. Red line depicts the Tn_4_Ca^2+^ structure and blue line depicts the Tn_2_Ca^2+^ structure. Tn_2_Ca^2+^ simulation was started from the equilibrated Tn_4_Ca^2+^ structure at 8 nanoseconds (marked with *).
(TIF)

Table S1 **List of systems, number and duration of simulations performed.**
(PDF)

Movie S1 **Hydrophobic core segment: PHE25, PHE74 and PHE77.**
(MPG)

Movie S2 **Expulsion of residue PHE28.**
(MPG)

Movie S3 **Movement of residues MET81, MET45 and MET80.**
(MPG)
