# Supplementary figures and images for: Calcium Induced Regulation of Skeletal Troponin — Computational Insights from Molecular Dynamics Simulations
Source: PLoS One. 2013 Mar 15;8(3):e58313. doi: 10.1371/journal.pone.0058313 (PMC3598806; doi:10.1371/journal.pone.0058313)

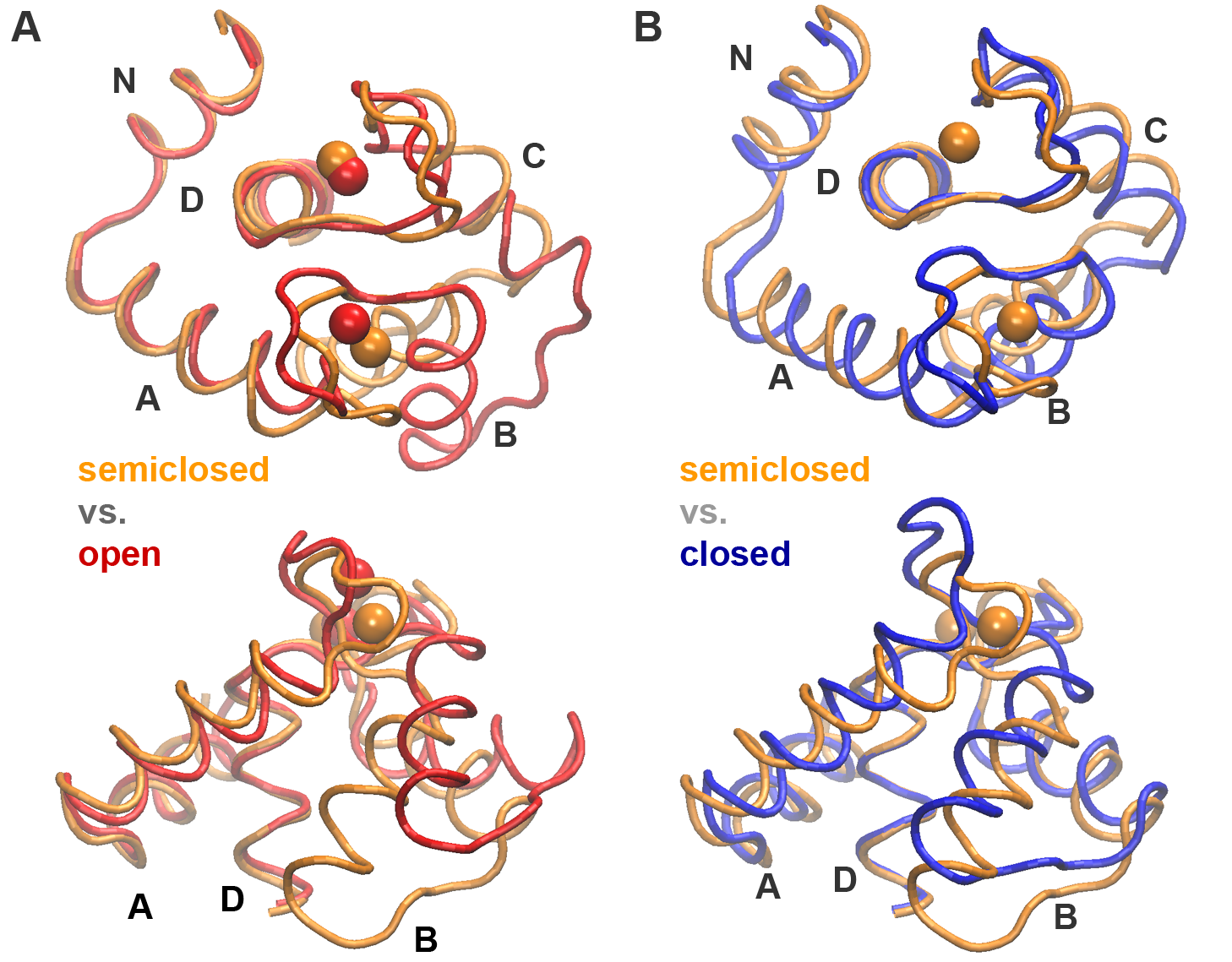

Supplement: Figure S1 — Open, semi-closed and closed states of TnC N-lobe. A. Structural comparison of the open and semi-closed states of TnC N-lobe. (shown in red and orange respectively). B. Structural comparison of the semi-closed and closed state of TnC N-lobe (shown in blue and orange, respectively). The helices of the N-lobe are labeled accordingly and the top panels show a ‘bird’s eye’ view of the N-lobe while the bottom panels show side views. The Ca2+ ions are shown as vdW shapes and in red and orange depending on if they are located in the open or semi-closed state structures. There are no Ca2+ ions in the closed state structures. (TIF) [file pone.0058313.s001.tif]

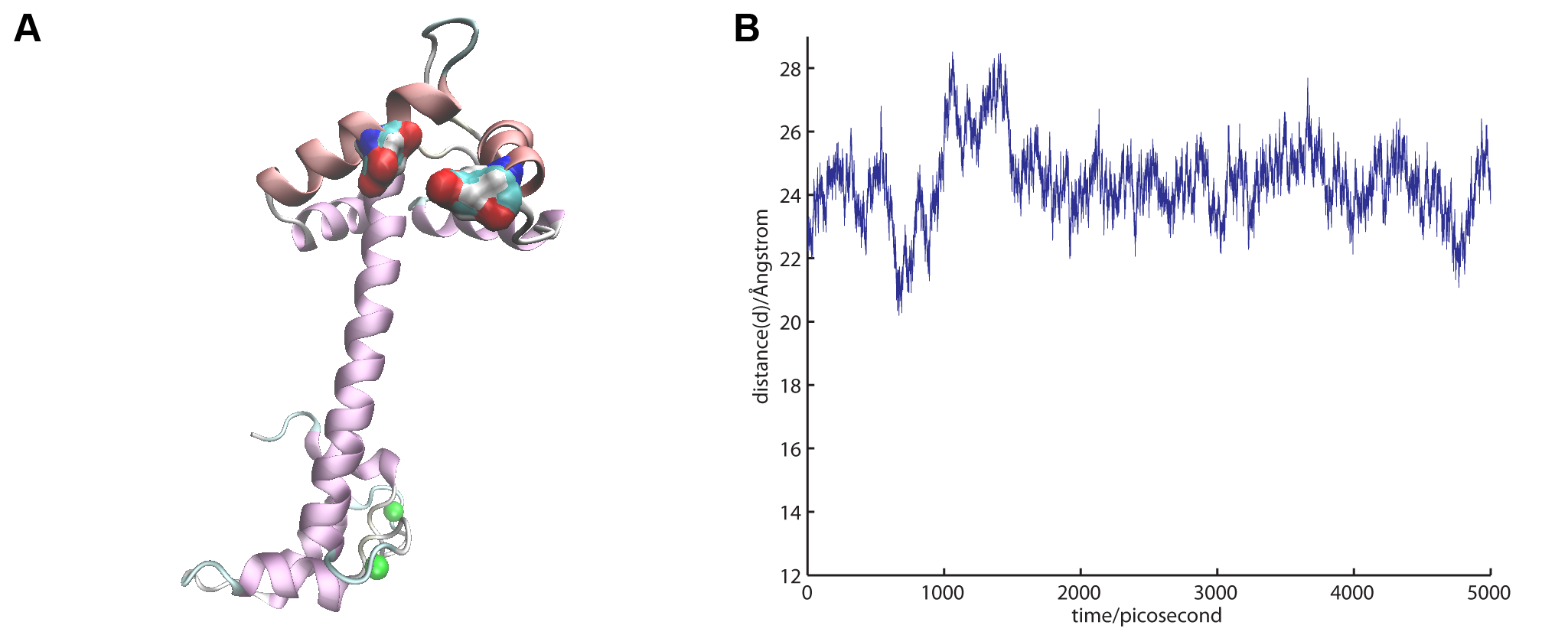

Supplement: Figure S2 — A mutation to prevent the TnC hydrophobic pocket from closure. A. Mutation of TnC N-lobe hydrophobic pocket residues ALA24 and LEU48 to ASP and GLU, respectively. The mutated residues are shown in surface representation and are seen protruding into the pocket. B. The evolution of the openness of the hydrophobic pocket of TnCASP24GLU48 in the Ca2+ depleted state as measured by the distance d between the alpha-C atoms of residues GLU16 and GLU48. (TIF) [file pone.0058313.s002.tif]

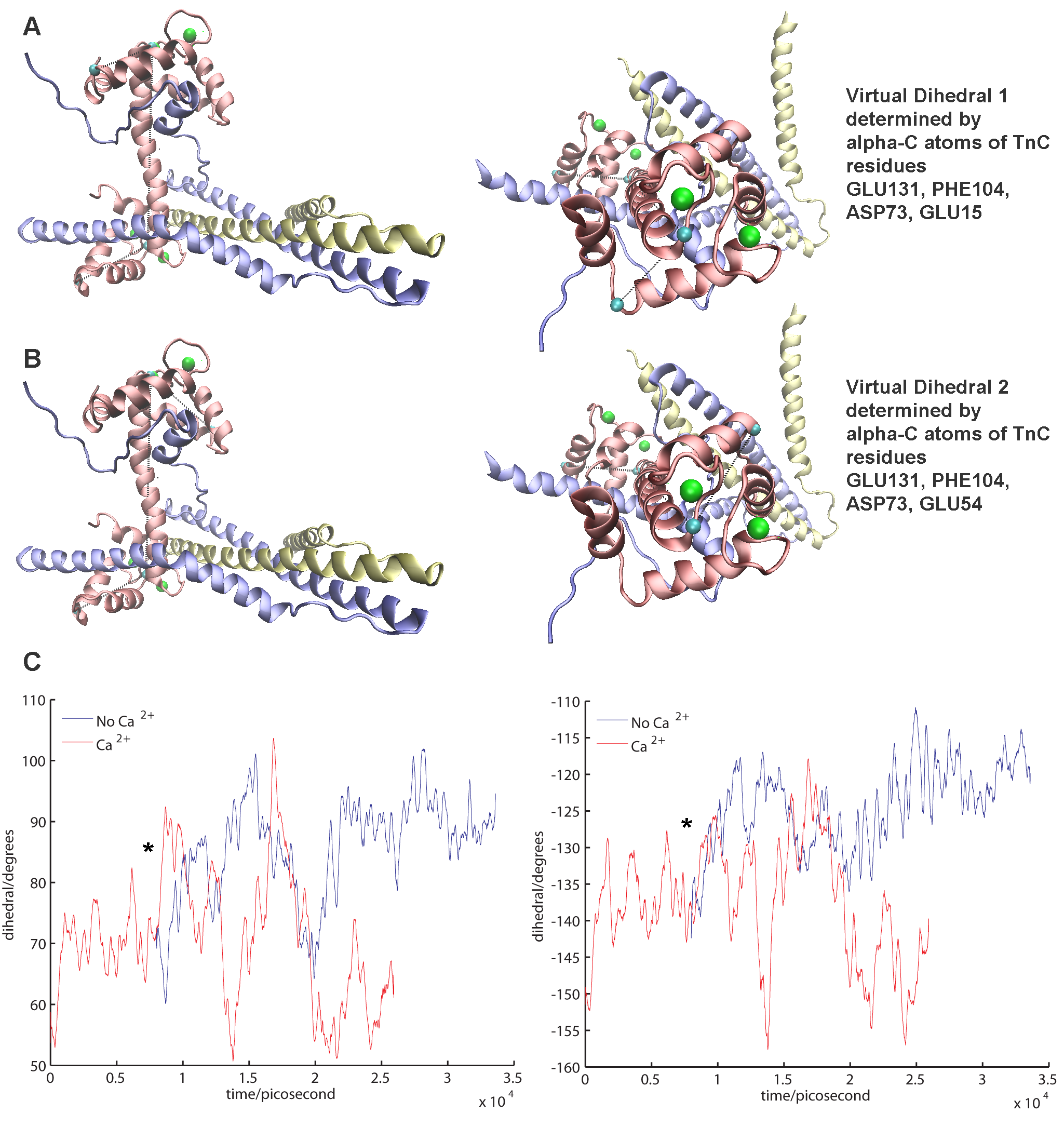

Supplement: Figure S3 — Rotation of the TnC N-lobe. A: Virtual dihedral 1 is determined by alpha-C atoms of residues GLU131, PHE104, ASP73, GLU15. The intersection of the two planes formed by alpha-C atoms of (GLU131, PHE104, ASP73) and alpha-C atoms of (PHE104, ASP73, GLU15) runs along the α-helix connecting the N-lobe and C-lobe of TnC. Residue 131 is located in the C-lobe of TnC. Residue 104 and 73 form a segment that spans the TnC connecting helix and Residue 15 is located at the start of the A helix. B: The virtual dihedral 2 is determined by alpha-C atoms of residues GLU131, PHE104, ASP73, GLU54. The intersection of the two planes formed by alpha-C atoms of (GLU131, PHE104, ASP73) and alpha-C atoms of (PHE104, ASP73, GLU54) runs along the α-helix connecting the N-lobe and C-lobe domains of TnC. Residue 131 is located in the C-terminal domain TnC. Residue 104 and 73 form a segment that spans the TnC connecting helix and Residue 54 is located at the start of the C helix. Alpha-C atoms of residues GLU131, PHE104, ASP73, GLU75, GLU15 and GLU54 of TnC shown as green vdW spheres. C. (Left panel) Time dependency of Virtual dihedral 1. Red line depicts the Tn4Ca2+ structure and blue line depicts theTn2Ca2+ structure. Tn2Ca2+ simulation was started from the equilibrated Tn4Ca2+ structure at 8 nanoseconds (marked with *). C: (Right panel) Time dependency of Virtual dihedral 2. Red line depicts the Tn4Ca2+ structure and blue line depicts the Tn2Ca2+ structure. Tn2Ca2+ simulation was started from the equilibrated Tn4Ca2+ structure at 8 nanoseconds (marked with *). (TIF) [file pone.0058313.s003.tif]
