# Supplementary material for: Calcium Induced Regulation of Skeletal Troponin — Computational Insights from Molecular Dynamics Simulations
Source: PLoS One. 2013 Mar 15;8(3):e58313. doi: 10.1371/journal.pone.0058313 (PMC3598806; doi:10.1371/journal.pone.0058313)
Supplement: Table S1 — List of systems, number and duration of simulations performed. (PDF) [file pone.0058313.s004.pdf]

SI Table 1.

System Name	based on PDB	# of sims.	Time/ns	Molecule
Tn_4Ca^{2+}	1YTZ	3	83	Tn complex Ca^{2+} saturated
Tn_2Ca^{2+}	1YTZ	3	94.4	Tn complex Ca^{2+} depleted
TnC_4Ca^{2+}	1YTZ	3	116	TnC Ca^{2+} saturated
TnC_2Ca^{2+}	1YTZ	3	120	TnC Ca^{2+} depleted
$TnCSite2noCa^{2+}$	1YTZ	1	20	TnC Ca^{2+} in site 1
$TnCSite1noCa^{2+}$	1YTZ	1	20	TnC Ca^{2+} in site 2
5TnC	5TnC	1	20	TnC Ca^{2+} depleted
TnC ASP24GLU48	1YTZ	1	5	TnC Ca^{2+} depleted
		16	478.4ns	
